# Retrospective Review of Blood Culture-Confirmed Cases of Enteric Fever in Navi Mumbai, India: 2014–2018

**DOI:** 10.4269/ajtmh.23-0102

**Published:** 2023-08-07

**Authors:** Niniya Jayaprasad, Priyanka Borhade, Christopher LeBoa, Kashmira Date, Shrikrishna Joshi, Rahul Shimpi, Jason R. Andrews, Stephen P. Luby, Seth A. Hoffman

**Affiliations:** ^1^ National Public Health Surveillance Project, World Health Organization–Country Office for India, New Delhi, India;; ^2^ Division of Infectious Diseases and Geographic Medicine, Department of Medicine, Stanford University School of Medicine, Stanford, California;; ^3^ Global Immunization Division, Center for Global Health, Centers for Disease Control and Prevention, Atlanta, Georgia;; ^4^ Dr. Joshi’s Central Clinical Microbiology Laboratory, Navi Mumbai, India

## Abstract

India has one of the highest estimated burdens of enteric fever globally. Prior to the implementation of Typbar-TCV typhoid conjugate vaccine (TCV) in a public sector pediatric immunization campaign in Navi Mumbai, India, we conducted a retrospective review of blood culture-confirmed cases of typhoid and paratyphoid fevers to estimate the local burden of disease. This review included all blood cultures processed at a central microbiology laboratory, serving multiple hospitals, in Navi Mumbai (January 2014–May 2018) that tested positive for either *Salmonella* Typhi or *Salmonella* Paratyphi A. Of 40,670 blood cultures analyzed, 1,309 (3.2%) were positive for *S.* Typhi (1,201 [92%]) or *S.* Paratyphi A (108 [8%]). Culture positivity was highest in the last months of the dry season (April–June). Our findings indicate a substantial burden of enteric fever in Navi Mumbai and support the importance of TCV immunization campaigns and improved water, sanitation, and hygiene.

Enteric fever is a disease caused by *Salmonella enterica* subspecies *enterica* serotype Typhi (*S*. Typhi) or Paratyphi A, B, or C (*S*. Paratyphi A, B, or C). Enteric fever caused an estimated 14 million cases and 136,000 deaths in 2017.^1^ The most cases occurred among children aged 5–9 years; 56% were in individuals of < 15 years.^1^ India accounted for the majority of the 2017 global burden, with an estimated 8 million cases (57%) and 72,000 deaths (53%).^1^ India also has one of the highest antibiotic utilization rates in the world,^2^ alongside worsening rates of antimicrobial resistant typhoid.^3^ In the setting of the increasing global prevalence of antimicrobial resistant typhoid, the World Health Organization (WHO) has prioritized typhoid vaccine delivery in conjunction with water, sanitation, and hygiene interventions.^4^

In 2017, the WHO prequalified a new typhoid conjugate vaccine (TCV), Typbar-TCV ^®^ (Bharat Biotech International Limited, Hyderabad, India) based on the *S*. Typhi Vi antigen.^4^ This vaccine can be used in children as young as 6 months old, has improved immunogenicity compared with prior typhoid vaccine generations, and has an expected longer duration of protection than unconjugated Vi antigen vaccines.^4^

In 2018, the Navi Mumbai Municipal Corporation (NMMC), the local governing body of Navi Mumbai, India, introduced the first public sector, pediatric TCV mass vaccination campaign.^5^ Navi Mumbai is located outside the industrial area of Greater Mumbai, the capital of Maharashtra State, and has a high burden of pediatric typhoid along with increasing prevalence of antimicrobial resistant typhoid isolate.^5^^,^^6^ Prior to the widescale implementation of Typbar-TCV in Navi Mumbai, we endeavored to understand the local burden of *S.* Typhi and *S.* Paratyphi infections.

We conducted a retrospective review of blood culture-confirmed cases of *S.* Typhi and *S.* Paratyphi A between January 2014 and May 2018. Data were obtained from Dr. Joshi’s Central Clinical Microbiology Laboratory (Joshi Laboratory), a privately run laboratory in Navi Mumbai, that serves many local healthcare facilities. We included all blood culture samples referred to Joshi Laboratory during the study period, which by default included evaluation for *S*. Typhi and *S.* Paratyphi A but not for *S.* Paratyphi B or C. All samples were submitted at the discretion of the patients’ primary providers, and only samples from within NMMC boundaries were evaluated.

Joshi Laboratory evaluated blood culture samples using an automated blood culture system (BD BACTEC^TM^). Further subculture was performed using sheep blood agar and MacConkey agar for differentiation of lactose-fermenting from non-lactose-fermenting gram-negative bacteria colonies. Conventional biochemical tests using oxidase, urease, indole, citrate, and triple-sugar iron (TSI) were performed to identify and confirm pathogens (*S.* Typhi: oxidase-negative, urease-negative, indole-negative, citrate-negative, TSI K/A/H_2_S-positive/gas-negative; *S.* Paratyphi: oxidase-negative, urease-negative, indole-negative, citrate-negative, TSI K/A/H_2_S-negative/gas-positive).

A deidentified spreadsheet was shared by Joshi Laboratory including month and year of blood culture and whether the blood culture was positive or negative for *S.* Typhi or *S.* Paratyphi A. A subset of positive blood cultures underwent antibiotic sensitivity testing, which included additional information about the participant (age and gender), but these antibiotic sensitivity testing results were excluded because of quality control concerns. Statistical analysis was performed in R (version 4.1.0). We evaluated the monthly blood culture *S.* Typhi and *S.* Paratyphi A positivity rate compared with monthly NMMC rainfall data^7^ and assessed sample correlations at lagged times (R version 4.1.0) to evaluate seasonal variation in the blood culture positivity rate based on rainfall amount. We used our positive cases and the Navi Mumbai 2011 census of ∼1.12 million persons^8^ to calculate crude typhoid and paratyphoid incidence rates (where complete yearly data were available from Joshi Laboratory; 2014–2017). Given the low sensitivity of blood culture (59%),^9^ we calculated adjusted incidences to account for missed cases ([crude incidence]*1/[1 − 0.59]).

Local institutional review board approval was not solicited because the study involved secondary data analysis, although the protocol for this study was approved by the Stanford University Institutional Review Board (eprotocol 39627). All data provided to investigators were deidentified.

A total of 40,670 blood cultures were tested for *S*. Typhi and *S*. Paratyphi A. Of these, 1,309 (3.2%) were positive. *S.* Typhi was the predominant isolate, accounting for 1,201 blood cultures (92%), whereas *S.* Paratyphi A accounted for 108 blood cultures (8%).

Of the 1,309 blood cultures positive for *S.* Typhi and *S.* Paratyphi A, gender information was available for 922 cases (377 females, 41%; 545 males, 59%) and of these, 701 cases included ages < 2 years (31, 4%), 2–5 years (133, 19%), > 5–15 years (349, 50%), > 15–45 years (175, 25%), and > 45 years (13, 2%) (Figure 1). The median enteric fever case age was 10 years for both males and females.

**Figure 1.**
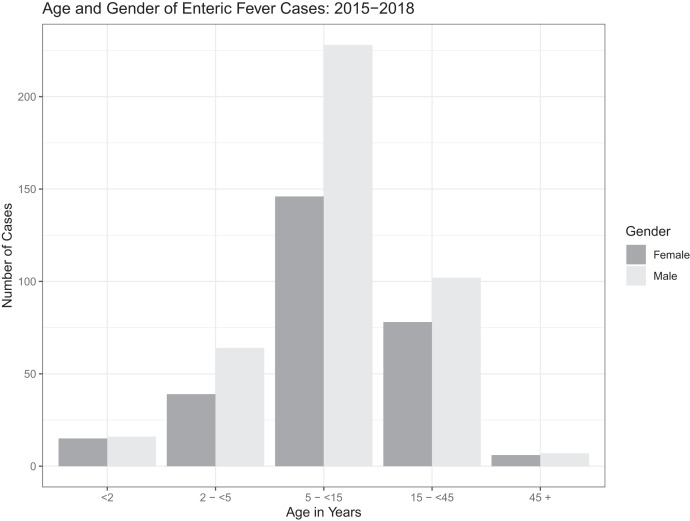
Breakdown of positive blood cultures by age and gender. Dark gray indicates female; light gray indicates male.

The proportion of blood cultures that grew either *S.* Typhi or *S.* Paratyphi A varied by year: 2014, 3.02%; 2015, 2.57%; 2016, 3.76%; 2017, 3.19%; and 2018, 4.16%. The peak proportion of blood cultures that grew either *S.* Typhi or *S.* Paratyphi A preceded peak rainfall over time by ∼2 months, suggesting an association between blood culture positivity and the terminal period of the dry season (Figure 2; dominant cross-correlation lag, +2; autocorrelation, 0.46; *P* < 0.05). The percent and raw number of specimens that grew either *S. typhi* or S. Paratyphi A varied by month (Figure 2).

**Figure 2.**
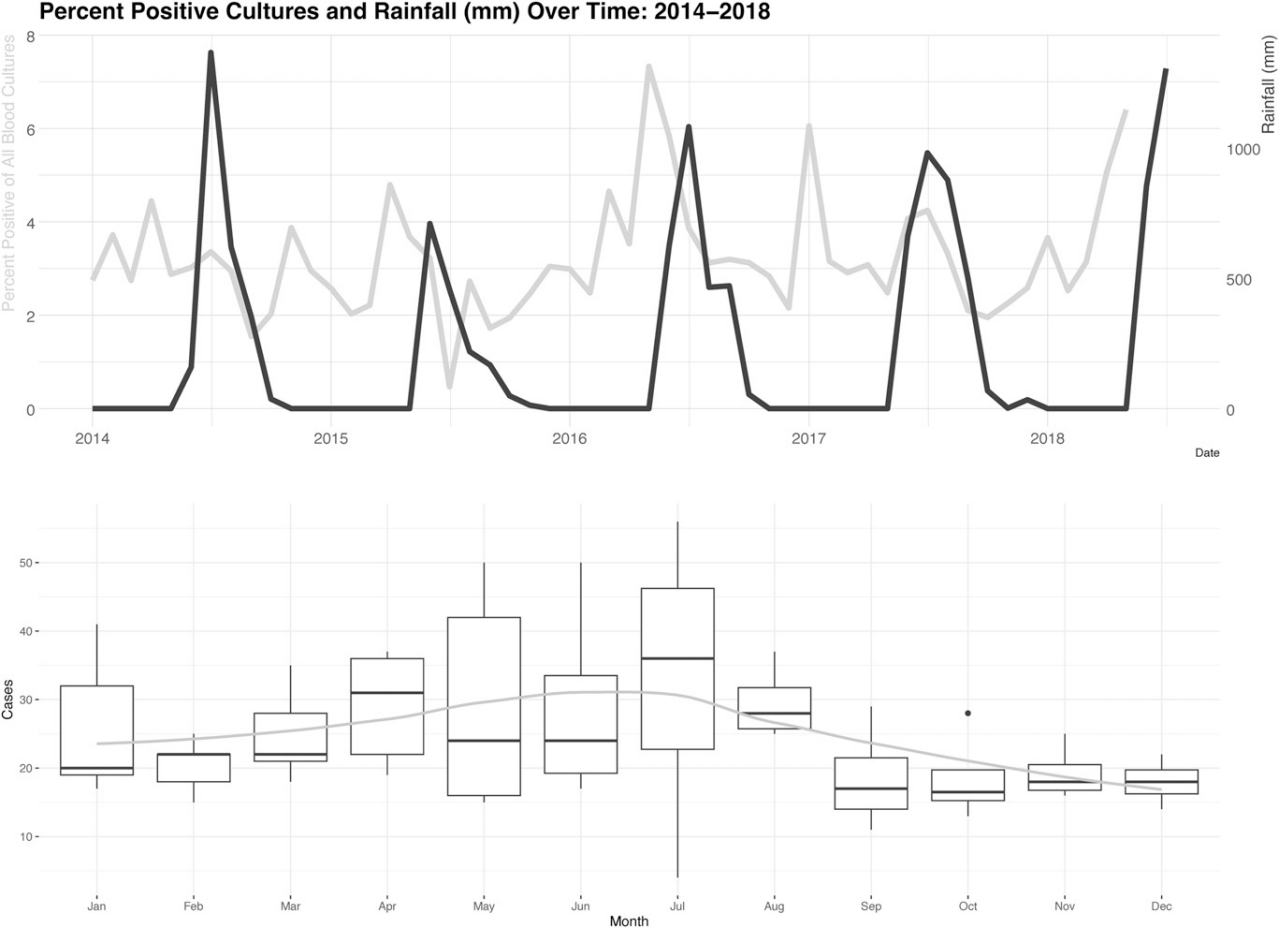
Top: Percent positive blood cultures and rainfall (mm) over time: 2014–2018. Blood culture positivity is shown in light gray; rainfall is shown in dark gray. Bottom: Case counts by month: 2014–2018. Smoothed conditional means are shown by the gray line.

The overall average *S.* Typhi and *S.* Paratyphi A positivity rate for blood cultures performed at Joshi Laboratory between 2014 and 2018 was 3.1%, which is similar to the rate reported from Vellore, India (3.8%) over a comparable time period.^10^ A retrospective review from the Surveillance for Enteric Fever in Asia Project portrayed a much lower enteric fever blood culture positivity rate (0.53%) from five hospitals across India.^11^ The enteric fever blood culture positivity rate at Joshi Laboratory trended up over the course of the study period, whereas other South Asian data suggest decreased rates from 2000 to 2015 in India, which has been attributed to improved economic factors, sanitation, and hygiene.^12^

The majority of cases were in males (59%), which is in line with overall sex disparities in enteric fever reporting in South Asia.^13^ For all study years, a spike in case positivity preceded the monsoon’s peak rainfall by ∼2 months, except in 2017, when the highest case positivity was in January (Figure 2). In contrast to prior data in which South Asian enteric fever case burden was described as highest during periods of peak rainfall or just after,^14^ our sample suggests an association with the dry season in Navi Mumbai. Similarly, in a community-based case-control study in Jakarta, Indonesia, there was a peak in enteric fever cases during the dry season compared with the wet season (ratio of 7:3).^15^ Depending on the source of water, during periods of water scarcity, such as the dry season, persons may be more prone to using contaminated water sources and/or the impact of a contaminant may be amplified, as has been described in cholera seasonality in South Asia.^16^

The calculated crude typhoid incidence was 23/100,000 and the paratyphoid incidence was 2/100,000. The adjusted typhoid incidence was 56/100,000 and the paratyphoid incidence was 5/100,000. These are likely underestimates for Navi Mumbai, given blood culture results are dependent on healthcare-seeking behavior, and South Asian data indicate only a minority of individuals in endemic settings receive blood cultures when febrile.^17^ Even as a minimum estimate from just one of Navi Mumbai’s laboratories, a typhoid incidence of 56/100,000 would be considered medium typhoid incidence.^18^ Our calculated crude incidence values are similar to recent South Asian data, in which adjusted incidences were calculated utilizing a hybrid surveillance scheme and projected 7–10 times greater burden than crude estimates.^17^ If applied to our crude estimates, this would suggest an adjusted *S.* Typhi incidence of 161/100,000 (i.e., [23/100,000]*7)—high typhoid incidence.^18^

We used culture positivity over time because prior work suggests this as a potential method of making inferences on typhoid fever incidence,^19^ although it can be confounded by other infectious diseases circulating in the community and may result in enteric fever burden underestimates. Our age and gender data are limited because only a fraction of our total dataset (age data: 922/1,309, 70%; age and gender data: 701/1,309, 54%) included this information, and thus may not accurately represent Navi Mumbai. Finally, we estimated crude incidence but used > 10-year-old census data, which would overestimate the crude incidence given presumed population growth since 2011. That said, blood cultures were collected at the primary provider’s discretion, which may result in missed nonsevere or atypical cases, and this underdiagnosis is compounded by the low sensitivity of optimally collected blood cultures (59%), made even lower with antibiotic use.^9^

This retrospective review of the results of blood cultures from just one of Navi Mumbai’s diagnostic laboratories is comparable to more complete data from throughout South Asia^10^^,^^17^ and indicates a medium typhoid burden in Navi Mumbai at a minimum. Our data support further work to investigate mechanisms by which Navi Mumbai’s enteric fever case positivity rate is highest during the dry season. These data support the importance the NMMC has placed on TCV immunization campaigns and highlight the continued importance of enteric fever surveillance and control, especially in light of the increasing prevalence of antimicrobial resistant typhoid fever in South Asia and globally.
